# Surgery of frontal sinus fractures. Epidemiologic study and evaluation of techniques

**DOI:** 10.1016/S1808-8694(15)30056-2

**Published:** 2015-10-19

**Authors:** Jair Cortez Montovani, Emanuel Araújo Nogueira, Fabricio Dominici Ferreira, Arlindo Cardoso Lima Neto, Victor Nakajima

**Affiliations:** a Associate Professor.; b Otorhinolaryngologist; c 3rd year resident of Otorhinolaryngology/head and neck surgery - University Hospital - Botucatu, Botucatu School of Medicine - UNESP; d 2nd year resident of Otorhinolaryngology/head and neck surgery - University Hospital - Botucatu, Botucatu School of Medicine - UNESP; e Assistant Professor of Otorhinolaryngology/head and neck surgery - University Hospital - Botucatu, Botucatu Medical School of Medicine - UNESP

**Keywords:** facial fracture, frontal sinus, surgical techniques

## Abstract

The frontal sinus trauma is not rare and it is 8% of the facial fractures. It can affect the anterior and/or posterior plates, with or without hitting the nasofrontal duct. It has a large potential of complications and its management still being a controversy. **Objective:** To present the casuistic of fractures frontal sinus, the epidemiology and clinical and surgical management of frontal sinus fractures. **Materials and Methods:** Not randomized retrospective study of 24 patients with frontal sinus fractures Hospital of Clinics, School of Medicine Botucatu, São Paulo, Brazil. **Results:** From the 24 patients, we had 16 (66,6%) fractures of the extern plate and 8 (33,4%) of both. In 2 patients the nasofrontal duct was involved. Others facial fractures were associated in 20 (83,4%) cases and major lesions of the cerebral segment were found in 13 (54,2%). Subpalpebral incision was performed in the majority with satisfactory aesthetic results. The basis of the surgical treatment was reduction and fixation with different materials (steel wire, mononylon, titanium miniplates) and if necessary we used alogen implants or parietal bone to reconstruct the anterior plate. **Conclusion:** The principal cause of frontal sinus fractures is crashed car. The management depends of the complexity, because commonly there are cranioencephalic lesions associated. The surgical thecniques used are the incisions, bicoronal flap or browglabella, infra-orbital rim (“butterfly”), associated a endoscopy sinus surgery in cases of infection, cerobrospinal fluid leak and orbital complications.

## INTRODUCTION

The frontal sinuses derive from the frontal recess, part of the middle meatus and the air cells of the ethmoidal infundibulum. Their aeration and development are radiologically evident at the ages of 5 or 6 years, and their full development will happen at the ages of 10 to 12 years. About 4% of the population does not have frontal sinuses and other 4 to 5% have only small upper air cells. A complete septum separates the right from the left frontal sinus, and these can be further divided in subcompartments or recesses by complete or incomplete bone septums^2^.

When developed, the frontal sinuses are located between the internal and external plates of the frontal bone, and both walls may be very thin. The anterior bone wall may be less resistant to impact forces, but it is somehow protected by the more prominent supraorbital contour, made up of high resistant bone^3^. The frontal sinuses are closely associated to the orbital roof, ethmoidal cells, nose and anterior cerebral fossa^3^, ^4^, ^5^, ^6^.

Differently from what many physicians think, frontal sinus injuries are not rare and correspond to 8% of all facial fractures^7^. Its etiology may vary according to the population studied, gender, age range and a person’s social, economical and cultural level.

Most frontal sinuses injuries are related to automobile accidents, physical aggressions, fire arm wounds and civil construction accidents. In 1987, Luce^7^ published a series of 78 cases, of which 61 had high speed automobile accidents as cause.

As to fracture type, the most common is the frontal sinus anterior plate, although the most severe cases also involve the posterior plate and/or the sinus floor, and the naso-frontal duct may be involved^8^, ^9^, ^10^. In less severe injuries, the anterior plate protects the posterior, and the former is usually affected alone. The great impact injuries affect both the plates and the floor with bone fragmentation and derrangement^11^, ^12^, ^13^.

By studying craniofacial trauma, Nahum^14^ showed that the impact force necessary to cause a frontal sinus fracture is of 360 to 990 Kg (800 to 2,2001b), what is enough to cause other head injuries. Depending on trauma intensity, there may be injuries on the anterior and posterior plates, and the latter is frequently associated to central nervous system, orbits and ethmoidal cell lesions^15^. Calvert (1942)^16^ described a series of 1,751 head trauma cases, of which 103 (15%) involved the frontal and ethmoid sinuses. 70% were compound fractures, of which 35% of the patients reported anosmia. Whigt et al. (1992)^2^ reported that 76% of their patients with anterior and posterior plate injuries had conscience alterations and 93% had multiple facial and cranial fractures.

A controversial aspect in these fractures is nasofrontal duct handling and the possibility of complications when it is damaged, such as sinusitis and frontal sinus mucocele. For some authors, the most common cause of frontal mucocele are frontal sinus and nasofrontal duct injuries^17^, ^18^. Others believe the nasofrontal duct obstruction in frontal sinus injuries is less frequent that what has been described in previous papers, thus changing so far established concepts as to the need for mucosal cauterization, curettage and even frontal sinus obliteration^17^, ^19^, ^20^.

Even with this concept review and its potential, frontal sinus complications still represent a dilemma for facial trauma surgeons, specially because they are rarely approached by multidisciplinary teams, and this brings about a great variation in handling and repair surgical techniques for these injuries^6^, ^15^, ^19^, ^21^. We must also bear in mind that many of the severe complications such as CSF fistulas and ocular damage may be present regardless of correct handling these injuries^11^, ^17^, ^19^, ^20^, ^21^, ^22^.

Our goal with this paper is to show our experience in caring for patients with frontal sinus fractures, discuss literature data and compare them to the approaches used in our facilities.

## MATERIALS AND METHODS

We carried out a non-randomized retrospective study with 24 patients diagnosed with frontal fracture, admitted to the Botucatu Medical School University Hospital - Department of Otolaryngology - Head and Neck Surgery, between January 1995 and December, 2004. The data was obtained through the analysis of their charts and the specialized care protocol of facial trauma. Failures in post-operative follow up or patient chart records were considered sample exclusion criteria.

We analyzed populational variables (gender, age, and color), injury etiology, use of alcoholic beverages, fracture site, associated craniofacial injuries, surgical technique employed, handling of the nasofrontal duct and post-operative complications. The definitive diagnosis was based on tomographic findings and post-operative complications. Lesions were classified according to involved site: posterior and anterior plate, comminuting and associated fractures.

## RESULTS

Of the 24 patients selected, 23 (95.8%) were males and 1 (4.2%) was female. There were 17 (70.8%) white, 5 (20.8%) brown and 2 (8.4%) black patients. As far as age is concerned, 5 (20.9%) were between 20 and 29 years, 11 (45.8%) between 30 and 39 years, 6 (25%) between 40 and 49 years and 2 (8.4%) between 50 and 59 years.

**Graph 1:**
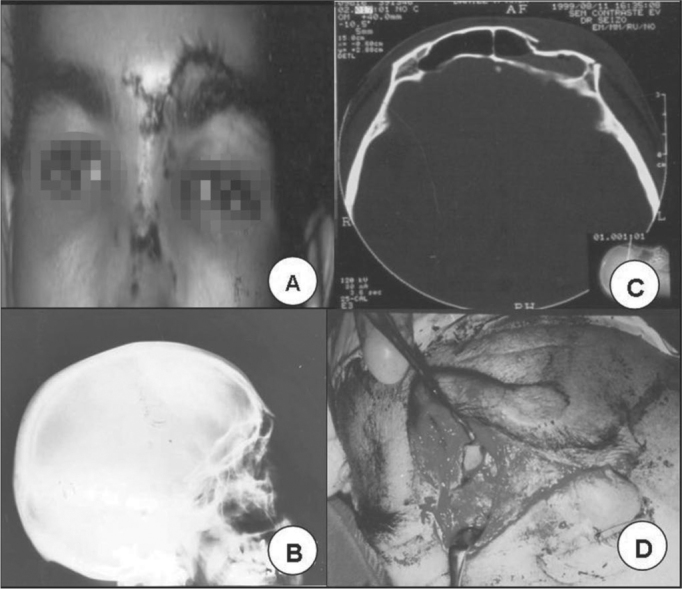
Etiology.

The most frequent etiology was that of automobile accidents, which occurred in 14 (58.3%) patients. In 16.7% the causes were fights, in 4 the cause was injury by objects and in 2 (8.4%) accidents with animals (falls and being kicked by a bull) (Graph 1).

In 10 (41.7%) there was past history of alcoholic beverage ingestion before the accident and in the remaining 14 (58.3%) there was no report of alcohol use or other drugs.

**Figure 1:**
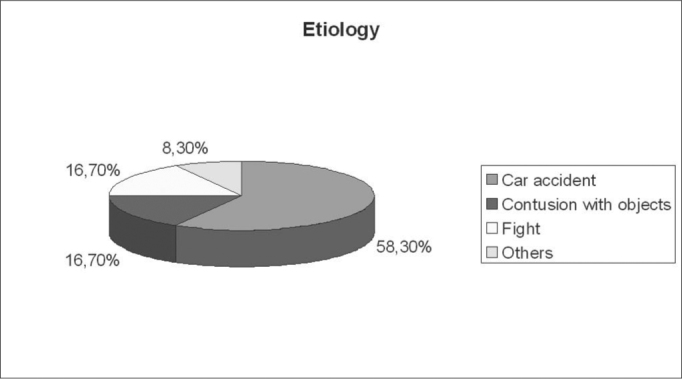
**A**. Patient with a cut and blunt injury on the left frontal region. **B**. skull lateral x-ray showing frontal sinus fracture. **C**. CT scan showing fracture with frontal sinus anterior wall sinking. **D**. Incision below the eyebrow showing frontal sinus anterior wall fracture.

**Figure 2:**
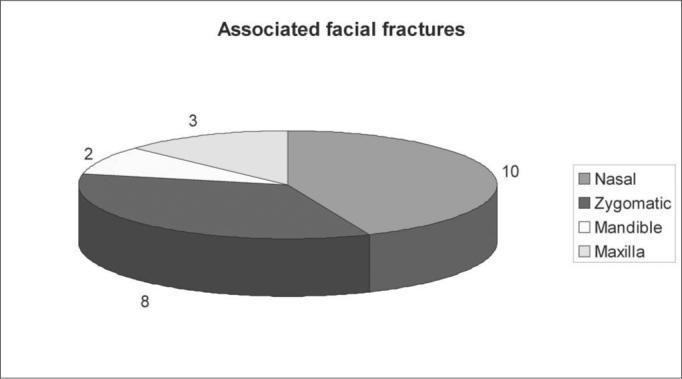
**A**. Facial sinus CT scan showing in A and B the frontal sinus posterior wall fracture with pneumoencephalus. **B**. Facial sinuses CT scan where we can see brain tissue herniation (meningocele) and pneumoencephalus.

As to the site of fracture, in 20 (83.4%) only the anterior plate was involved (Figure 1B and 1C) and in 4 (16.6%) there was fracture in both the anterior and posterior plates (Figures 2A and 2B). In 2 (8.4%) of these patients, there was injury in the nasofrontal duct. Exposed fractures were seen in 5 (20.8%) patients.

**Graph 2:**
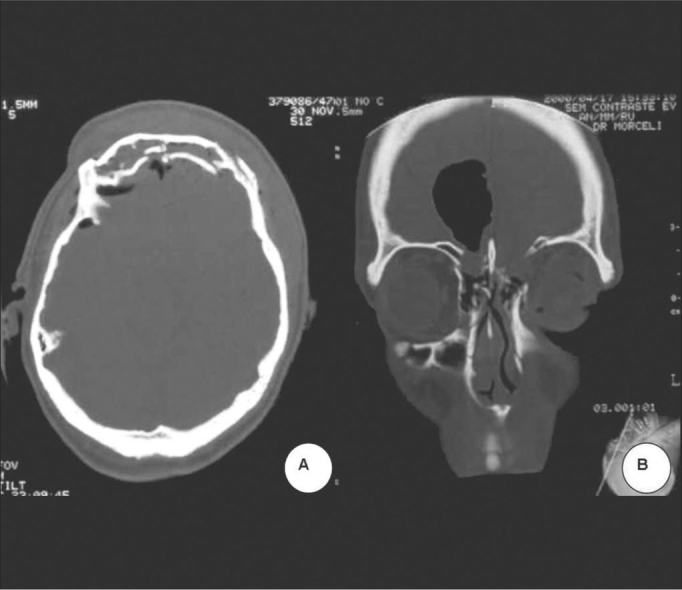
Associated facial fractures.

In 20 patients other facial fractures were seen - in 4 there were two fractures of facial bones, making up a total of 23 fractures: 10 (41.7%) nasal fractures, 8 (33.3%) zygomatic, 3 (12.5%) maxillas and 2 (8.4%) mandible (Graph 2).

**Graph 3:**
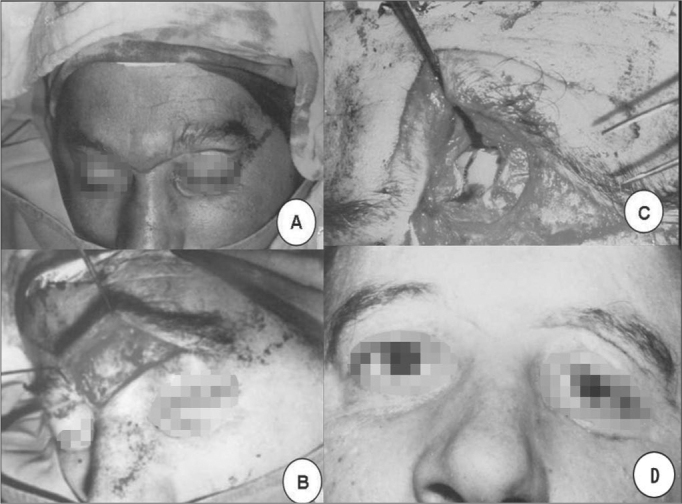
Associated head lesions.

**Figure 4:**
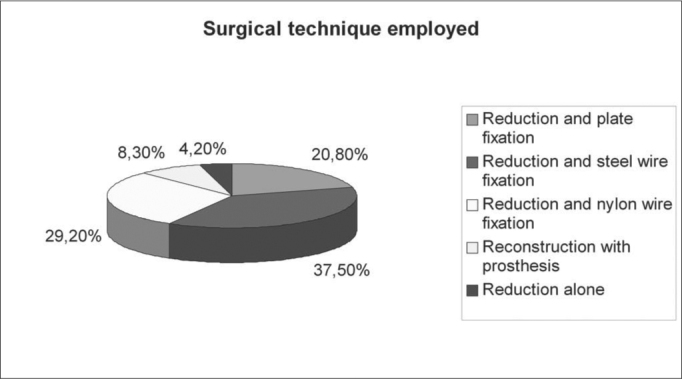
**A**. Frontonasal duct catheterization. **B**. Bone graft to rebuild the orbit roof. **C**. Silastic placement. **D**. Final aspect.

In most of the cases there was an association between the frontal sinus injury and important injuries in the cranioencephalic segment (54.2%) (Graph 3). The most common injuries were, in descending order: 5 patients (20.9%) intracranial hemorrhages, 4 (16.7%) pneumoencephalus, 3 (12.5%) CSF fistula, 3 (12.5%) skull base fracture and in one (4.2%) there was optical nerve compression (Figure 4).

**Figure 3:**
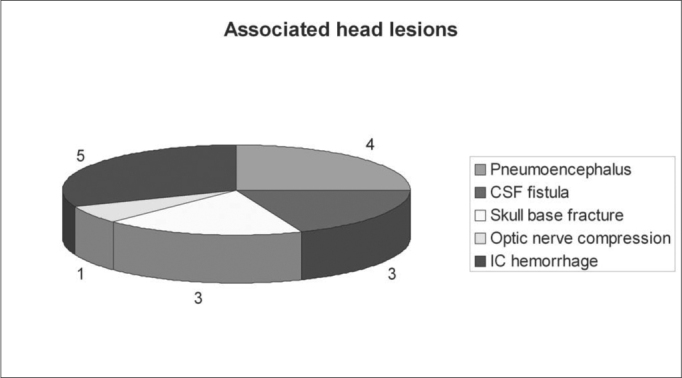
Below the eyebrow incision in butterfly shape and frontal sinus anterior wall fracture exposure.

**Graph 4:**
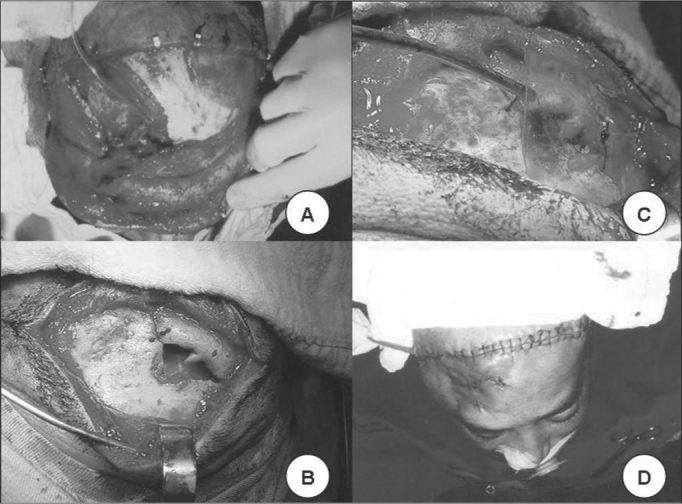
Surgical technique employed.

Treatment in all patients consisted of open reduction and bone fixation with different types of material. In 2 (8.4%) a bicoronal incision was made and in the others a butterfly wing-type of incision was made, bellow the eyebrow or, in cases of exposed fracture, through the wound itself (Figure 3). 2 patients with nasofrontal injuries were catheterized during the procedure (Figure 4). In order to fixate the bone fragments, a steel wire was used in 9 (37.5%), nylon monofilament wire in 7 (29.2%) and mini titanium fixation plates in 5 (20.8%) (Graph 4). In 2 (8.3%) implants were used in order to reconstruct the anterior wall, 1 Teflon and the other was porous polyethylene. In 3 other patients, a parietal bone graft was used to reconstruct the frontal plate associated to the use of Teflon (silastic) (Figures 4B and 4C). In only one patient, it was possible to reduce the bone fragments without the need for fixation (Figures 3B and 3C). In the patient with optical nerve compression we carried out endoscopic decompression.

Post operative complications or those accruing from the trauma itself were: porous polyethylene implant extrusion after 9 months of follow up in 1 patient; 2 (8.3%) patients had anosmia associated to the anterior skull base fracture; one patient had mucocele formation, which was solved by FESS 6 months after the initial injury.

## DISCUSSION

The etiology behind our cases was similar to those found in the literature, in other words, automobile accidents and fights. In our diagnostic assessment we used an easy to apply classification (anterior and posterior plates of the frontal sinus, comminuting fracture and related injuries), suggested by Luce (1987)^7^ and Donald (1982)^18^. Although being simple, this classification allows us to separate the cases with the greatest potential for complications (posterior plate lesions), which require more complex surgical procedures and medical care, from those which may be managed through simpler surgical approaches, such as the fractures involving the anterior plate of the frontal sinus^3^, ^11^, ^15^, ^21^, ^20^.

We believe that clinical observation, supported by ancillary exams (endoscopy, radiographies, CT scans and MRI) shall guide the treatment. The most common of these approaches are: impressions written down by the first attending physician to see the patient, vital signs, level of conscience, hemorrhages and soft tissue involvement, including frontal bone sinking, supra-orbitary anesthesia and conjunctival echimosis. Frequently omitted data are eye ball injuries and sero-bloody nasal secretion indicating a possible nasal CSF fistula.

Although today nasal endoscopy and CT scan are considered fundamental to diagnose frontal sinus fractures, we believe that the simple x-ray in the postero-anterior, lateral, Waters and Caldweel views are still useful for a first assessment of these injuries, and what is even more important, they are available in almost all hospitals^3^, ^4^. However, when a lesion in the posterior plate and dura matter are suspected, data obtained through simple x-rays are not very reliable. In such cases, there is the need to order nasal endoscopy and CT scan of the skull and paranasal sinuses. Still, if possible, MRI and technetium scintigraphy to diagnose, for instance, a CSF leak^4^, ^7^, ^8^, ^12^, ^13^.

The opinion of many authors is that the time to operate a patient with frontal sinus fracture is still controversial. Rohrich and Hollier showed that surgery immediately after the injury reduces both morbidity and morbidity^15^. Our experience is the same; however, in many cases we have seen that morbidity or longer hospital stay was determined more by factors such as other body injuries than the delay in performing surgery. We must bear in mind that many of our patients have been seen in other hospitals and, only after days, were referred to our service, and this surely delayed treatment. In fracture cases and when there are cranioencephalic complications, we recommend conservative measures such as the prescription of antibiotics that trespasses the blood-brain barrier, the use of intra-vascular expander (dextran) and 30° head elevation until proper diagnosis and management^8^, ^13^, ^21^. In severe lesions, such as CSF fistulas communicating the brain with potentially contaminated cavities (nose), lesions in the orbital cone and optical nerve, the patients should be operated upon in the first 72 hours after the injury, since the early diagnosis and treatment of these complications reduce both morbidity and mortality^11^, ^12^, ^13^, ^18^, ^19^, ^23^. Even then, it is not uncommon for these patients to develop meningitis, fistulas, intrarrhachidian abscesses, longer hospital stay and the need for two or more surgeries^17^, ^18^, ^22^. Another common sequel in these cases depends on the type of injury (cribiform plate fracture) or the type of surgery, which is permanent anosmia^10^, ^18^.

Another controversial issue in the literature is the pathway used to approach the frontal sinus fracture. In those simple fractures involving mainly the anterior wall we prefer to make our incision below the eyebrow instead of using bicoronal incision^15^, ^16^, ^21^, ^22^, ^24^. The latter, advocated by Snow and Parsons^19^, we performed in two cases and when using it, there is the possibility of forming ugly scars because of male bald pattern and, even the possibility of accelerating it^5^, ^15^, ^17^. Now, the “Butterfly Wing”-type of incision, below the eyebrow was the one most used by us. There were no apparent ugly scars in the face, because we made small incisions, following the lines of tension and using non-traumatic suturing techniques (intra-dermal sutures). The literature reports persistent hyperesthesia in the glabella and frontal region caused by injury to the supra-orbitary nerves, however we did not have it. We believe this might have been so because we were very careful in detaching and elevating the muscle-skin flap^7^, ^8^, ^15^, ^21^.

Compound or unstable fracture reduction and fixation with steel wires, very much used by us until the 80’s, has been increasingly abandoned, although it presented good cosmetic and functional results^15^. We currently use mononylon or mini titanium plates, being careful to bring the fragments as close as possible to each other and, thus, have a better facial appearance^6^, ^7^, ^8^. Notwithstanding, some patients complain they can feel both steel wires and mini plates as they touch their faces, thus requiring their removal after bone consolidation.

In the last 15 years, in many of the frontal sinus fractures and their complications, such as CSF fistulas, injuries to the frontonasal duct, mucopyoceles and orbit complications, the nasal endoscopic techniques associated or not to the open techniques are being increasingly indicated by otorhinolaryngologists^10^, ^15^, ^19^, ^23^, ^25^, ^26^. The latter, just as the open approach, allow the use of muscle fascia or nasal and oral mucosa free grafts, or evens the use of aloplastic material to correct dura mater defects^11^, ^12^, ^13^, ^17^, ^18^, ^20^.

Even today, the use of more aggressive techniques such as packing, obliteration and cranialization of the frontal sinus generate controversies^10^, ^17^, ^20^, ^18^, ^25^, ^27^. A number of papers show different approaches, varying from conservative measures, all the way up to aggressive surgery, especially in cases of intracranial and orbit complications^11^, ^12^, ^13^, ^17^, ^19^, ^22^, ^23^. Some of them, very much debated, are based on total nasal mucosa exeresis (curettage)^5^, ^6^, ^7^, ^8^, frontonasal duct obliteration^18^, ^20^, ^27^ and sinus packing with autogenous fat^17^, inducing osteogenesis.; and bear advantages over more conservative techniques^23^. Our experience shows that more conservative approaches must prevail over aggressive techniques. Only one of our patients required obliteration and packing of the frontal sinus with fat tissue and reconstruction of the nasal walls with bio-absorbable material (porous polyethylene and silicone). Notwithstanding, his procedure was an exception, because the patient had osteomyelitis and bone sequester in the frontal sinus after late referral (4 weeks) to our service. Techniques such as Rydell’s procedure, frontal sinus ablation, are rarely performed, because they may cause gross facial deformation^7^, ^11^, ^12^, ^20^, ^25^, ^26^.

Therefore, we may state that the decision about the best time to treat and the technique employed will depend on lesion severity and extension, and a full clinical assessment of both the patient and the injury. The procedure of choice in simple and isolated injuries should be the less aggressive possible and be based on the exploration and cleaning of the surgical wound, in observing the permeability of the frontonasal duct, internal fixation of bone fragments and cosmetic appearance. In these and in more extensive and severe lesions, with intracranial involvement, the use of many associated surgical techniques is more effective and adequate, among them we mention the nasal endoscopic approach. Sinus ablation, cranialization and obliteration are procedures with increasingly more restrictive indications.

## References

[2] Hungria H (2000). Otorrinolaringologia.

[3] Rowe NL, Killey HC (1969). Fractures of the facial skeleton.

[4] Hybels RL, Weimert TA (1979). Evaluation of Frontal Sinus Fractures. Arch Otolaryngol.

[5] Montgomery MW (1971). Surgery of the frontal sinus. Otolaryngol Clin North Am.

[6] Lee TT, Ratzker PA, Galarza M, Villanueva PA (1998). Early combined management of frontal sinus and orbital and facial fractures. J Trauma.

[7] Luce EA (1987). Frontal Sinus Fractures: Guidelines to Management. Plast. Reconstr Surg.

[8] Adkins WY, Cassone RD, Putsey FJ (1979). Solitary frontal sinus fractures. Laryngoscope.

[9] Stanley R.B. (1989). Fractures of the frontal sinus. Clin Plast Surg.

[10] Heller EM, Jacobs JB, Holliday RA (1989). Evaluation of the frontonasal duct in frontal sinus fractures. J Head Neck Surg.

[11] Donald PJ, Bernstein L (1978). Compound frontal sinus injuries with intracranial penetration. Laryngoscope.

[12] Hybels RL, Newman MH (1977). Posterior table fractures of the frontal sinus: I. Experimental study. Laryngoscope.

[13] Hybels RL, Newman MH (1977). Posterior table fractures of the frontal sinus: II. Clinical aspects. Laryngoscope.

[14] Nahum AM (1975). The Biomechanics of Maxillofacial Trauma. Clin Plast Surg.

[15] Rohrich RJ, Hollier LH (1992). Management of frontal sinus fractures. Changing concepts. Clin Plast Surg.

[16] Calvert CA, Cavins H. (1942). Injuries of frontal and etmoidal sinuses. Proc Roy Soc Med.

[17] Larrobee WF, Teavis LW, Tabl HG (1980). Frontal sinus fractures: their suppurative complications and surgical management. Laryngoscope.

[18] Wilson BC, Davidson B, Corey JP, Haydon RC (1988). 3rd. Comparison of complications following frontal sinus fractures managed with exploration with or without obliteration over 10 years. Laryngoscope.

[19] Reardon EJ (2002). Navigation risk associated with sinus surgery and the clinical effects of implementary a navigational system for sinus surgery. Laryngoscope.

[20] Bosley WR (1970). Oteoplastic obliteration of the frontal sinuses. Laryngoscope.

[21] Helmy ES, Koh ML, Bays RA (1990). Management of frontal sinus fractures: review of the literature and clinical update. Oral Surg Oral Med Oral Pathol.

[22] Shumrick KA, Kertin RC, Kulmin DR, Sinha PK, Smith TI (1992). Extended access internal approaches for the management of facial trauma. Arch Otolaryngol Head Neck Surg.

[23] May M, Levine HL, Mester SJ, Schaitkin B. (1994). Complications of endoscopic sinus surgery. Analysis of 2018 patients - Incidence and Prevention. Laryngoscope.

[24] Snow RC, Parsons RW (1975). Exposure through a coronal incision for initial treatment of facial fractures. Plast Reconstr Surg.

[25] May M, Ogura JH, Schramm V (1970). Nasofrontal duct in frontal sinus fractures. Arch Otolaryng.

[26] Smith T, Han JK, Loehrl TA, Rhee JS (2002). Endoscopic Management of the Frontal Recess in Frontal Sinus Fractures: A Shift in Paradigm?. Laryngoscope.

[27] Donald PJ (1982). Frontal Sinus Ablation by Cranialization. Arch Otolaryngol.

